# Reduced burnout in medical and health science students during the pandemic COVID-19 - a follow-up study of a single institution in Hungary

**DOI:** 10.1186/s12909-023-04867-0

**Published:** 2023-11-22

**Authors:** David Sipos, Anett Anna Biro, Flora Busa, Omar Freihat, József Tollár, Attila András Pandur, Árpád Kovács, Krisztina Deutsch, Melinda Petőné Csima

**Affiliations:** 1 Department of Medical Imaging, Faculty of Health Sciences, University of Pécs, Szent Imre street 14/B, Kaposvár, 7400 Hungary; 2 Present Address: Radiation Oncology, Research, and Teaching Center, Dr. József Baka Diagnostic, “Moritz Kaposi” Teaching Hospital, Guba Sándor street 40, Kaposvár, 7400 Hungary; 3 College of Health Science, Abu Dhabi University, Department of Public Health, Abu Dhabi, United Arab Emirates; 4 Department of Oxyology, Emergency Care, Faculty of Health Sciences, University of Pécs, Vörösmarty 4, Pécs, 7621 Hungary; 5 Department of Oncoradiology, Faculty of Medicine, University of Debrecen, Nagyerdei 98, Debrecen, 4032 Hungary; 6 Institute of Emergency Care and Pedagogy of Health, Faculty of Health Sciences, University of Pécs, Vörösmarty Street 4, Pécs, 7621 Hungary; 7 Institute of Education, MATE - Hungarian University of Agriculture and Life Sciences, Guba Sándor street 40, Kaposvár, 7400 Hungary

**Keywords:** Student, Burnout, COVID-19, Secondment, Maslach burnout inventory general survey for students scale

## Abstract

**Background:**

The coronavirus pandemic has significantly impacted lives worldwide, especially of medical and health science students. In Hungary, education has been relegated to the online space, with a substantial proportion of students having to attend medical secondments. Increased stress, uncertainty, and the presence of medical secondments can have an impact on students’ premature burnout.

**Methods:**

In 2021, we conducted a follow-up survey among students of the University of Pécs studying medicine and health sciences in two data collection periods (from March to May and September to November). Our online questionnaire consisted of the Maslach Burnout Inventory General Survey for Students and our self-designed questionnaire. We used descriptive and paired two-sample t-tests for data analysis at a 95% confidence interval (*p* ≤ 0.05).

**Results:**

We excluded from our survey respondents whose data we could not follow-up; finally, 183 students’ responses were analyzed. The majority of students were female (*n* = 148; 80.9%). Overall, there was a significant decrease in both exhaustion (EX) and cynicism (CY) scores (*p* = 0.001; *p* = 0.004). Female respondents had higher EX scores, but a significant decrease was observed for both genders (*p* ≤ 0.05). Excluding paramedic students, a significant decrease in EX scores was observed for the specialties we studied (*p* ≤ 0.05). General medicine students’ CY scores decreased; physiotherapy students’ profesisonal efficacy (PE) scores increased significantly (*p* ≤ 0.05). Students who were on medical secondments (*n* = 127; 69. 4%) were found to be more affected by burnout, but in all cases, these scores significantly improved (*p* ≤ 0.05). Students serving in the National Ambulance Service (*n* = 76; 41.5%), Hospitals (*n* = 44; 24.0%), or both (*n* = 7; 3.8%) had a significant decrease in their burnout score (*p* ≤ 0.05). Students who served in either a hospital or a hospital and National Ambulance Service had significantly improved CY and PE scores (*p* ≤ 0.05). Students concerned about their health had elevated EX and CY scores, which also improved (*p* ≤ 0.05).

**Conclusions:**

In conclusion, medical secondments positively affected student burnout scores for medicine and health sciences students at our institution. This fact implies that it is necessary to have more internships in real-life settings during the training.

**Trial registration:**

Our survey has been approved by the Medical Research Council (Case No IV/4573-1/2021/ECU).

## Background

The COVID-19 (coronavirus disease 2019) pandemic tested the performance of healthcare systems, in which context the organization of practices in medical and health sciences courses in a hospital environment was challenging [1, 2]. As a result of the rapid spread of the pandemic in Hungary, critical education decisions had to be taken quickly, which resulted in the entire education system being brought online [3, 4]. Following the guidelines issued, the clinical rotation of medical and health science students, among others, has been temporarily suspended to give decision-makers time to gather more information on the risks and course of COVID-19 and to prepare for future safe work [2, 5–7].

Medical students have been shown to have a higher prevalence of mental health problems than the general population, including generalized anxiety disorders, depression, and burnout, even before the COVID-19 pandemic [8, 9]. Documented causes of stressors in medical students include increased study load, peer competition, work-life balance, family expectations, financial difficulties, and exposure to human suffering [8–11].

The international literature suggests that the vast majority of medical students feel an ethical obligation to participate in the pandemic management and are willing to fight against the current COVID-19 crisis, even if they consider the risk of infection to be high [12, 13].

In Hungary, medical and health sciences students were on so-called medical secondments. The students were involved in COVID-19 testing of the population, assisting in hospital work, and helping the National Ambulance Service [14].

## Methods

### Aim

While the impact of the COVID-19 pandemic on healthcare workers is well documented, in Hungary, to the best of our knowledge, the impact of the COVID-19 crisis on the mental health of students studying medicine and health sciences has yet to be adequately investigated. Our research aimed to assess the burnout of students studying medicine and health sciences at the University of Pécs and investigate the impact of medical secondments and aspects of the coronavirus on students’ mental health.

We conducted a quantitative, descriptive, follow-up study among the students of the Faculty of Medicine and the Faculty of Health Sciences of the University of Pécs.

Data collection took place at two points in time. The first data collection took place from March to May 2021, and the second from September to November 2021, taking care to minimize the impact of exam season pressure on our results. Our questionnaires were distributed to students as a university newsletter during both data collection periods and shared on social media platforms closely linked to the faculties participating in the survey.

### Pairing student responses

Before completing the questionnaire, students were asked to create an eight-character unique identification code to pair their responses to the questionnaires completed during the two data collection periods. The code consisted of the first and last letters of the student’s first name, the first and last letters of the student’s mother’s first name, the last two digits of the student’s birth year, and the date of birth.

### Applied questionnaires

#### Maslach burnout inventory – general survey for students

The Hungarian-validated version of the Maslach Burnout Inventory – General Survey for Students (MBI-GS (S)) was used to assess burnout. The MBI-GS (S) is a 16-question questionnaire divided into three subscales: exhaustion (five items), cynicism (five items), and professional efficacy (six items). Each item is rated on a seven-point Likert scale ranging from never (0) to every day (6) [15].

In the three-dimensional model, low levels of burnout were defined in line with the work of Costa et al. (2012) using the following scores: exhaustion (0–9), cynicism (0–1), and professional efficacy > 27. Moderate level was identified as following: exhaustion (10–14), cynicism (2–6), and professional efficacy (23–27). A high level of burnout was defined as exhaustion > 14, cynicism > 6, and professional efficacy < 23 [16].

High scores on the Exhaustion (EX) and Cynicism (CY) subscales and low scores on the Professional Efficacy (PE) subscale indicate burnout [16, 17].

### Self-designed questionnaire

In addition to sociodemographic characteristics, our questionnaire asked students about their experiences during the COVID-19 outbreak and the characteristics of their education through closed, simple multiple-choice questions.

### Ethical compliance

Before completing the anonymous questionnaire, the students were informed about the procedure and content of the survey and filled in a consent form. They could stop the survey at any stage of the questionnaire without giving any reason. Our survey has been approved by the Medical Research Council (Case No IV/4573-1/2021/ECU).

### Statistical analysis

Statistical data analysis was performed in SPSS (Statistical Package for the Social Sciences) version 23.0. The statistical test used was a descriptive, paired two-sample t-test at a 95% confidence interval (*p* ≤ 0.05).

## Results

### Results of the descriptive statistics

After data cleaning, 183 student responses were analyzed, with an average age of 22.8 years (SD = 5.16).

In terms of gender, female respondents (*n* = 148; 80.9%), and in terms of specializations, the highest numbers of students were studying to become general practitioners (*n* = 44; 24.0%), physiotherapists (*n* = 41; 22.4%), radiographers (*n* = 35; 19.1%) and nurses (*n* = 28; 15.3%).

At the time of our survey, 69.4% of respondents (*n* = 127) had been on a medical secondment. Most seconded respondents worked in the National Ambulance Service (*n* = 76; 41.5%) and hospitals (*n* = 44; 24.0%).

8.7% of the responding students (*n* = 16) had been infected with the coronavirus, and a further 25.1% (*n* = 46) had been subject to quarantine. Health concern was prevalent among 69.4% (*n* = 127) of university students.

### Prevalence of burnout among students studying medicine and health sciences

At the first data collection date, the MBI-SS internal consistency values for the dimensions of EX, CY, and PE were 0.913, 0.891, and 0.824, respectively. At the second data collection date, these values were 0.865, 0.823, and 0.825.

The values of EX and CY decreased significantly between the two data collection dates (12.43 vs. 10.52 (*t* = 5,821; *p* = 0.001); 6.89 vs. 5.93 (*t* = 2,902; *p* = 0.004)). No significant increase of PE score were observed between the two data collection dates (22.10 vs. 22.51 (*t*=-1,148; *p* = 0.252)) (Table 1).

**Table 1:** Table The results of the maslach burnout inventory general survey for students dimensions for the two sampling periods

	Maslach Burnout Inventory General Survey for Students					
	PRE Exhaustion	POST Exhaustion	PRE Cynicism	POST Cynicism	PRE Professional Efficacy	POST Professional Efficacy
Mean (SD)	12,43 (7,53)	10,52 (6,08)	6,89 (5,97)	5,93 (5,05)	22,10 (6,08)	22,51 (5,59)
Cronbach’s L	0,913	0,865	0,891	0,823	0,824	0,825
*t*, *p*	5,821; **0,001**		2,902; **0,004**		-1,148; 0,252	

Categorizing the results of the MBI-SS dimensions, in the EX dimension, the proportion of students in the low burnout category (44.8% vs. 53.6%) increased and the proportion of students in the moderate EX category (27.3% vs. 17.5%) decreased significantly in the second phase of the survey.

On the CY dimension, the proportion of students in the moderate category increased (31.0% vs. 36.1%), while the proportion of students in the high category decreased (45.9% vs. 43.2%).

Looking at the PE dimension scores, the proportion of students in the low category decreased significantly (53.6% vs. 26.8%), while the proportion of students in the high category increased significantly (29.0% vs. 56.3%) (Table 2).

**Table 2:** Table Categorical distribution of the maslach burnout inventory general survey for students dimensions by frequency and percentage

	Maslach Burnout Inventory General Survey for Students					
	PRE Exhaustion	POST Exhaustion	PRE Cynicism	POST Cynicism	PRE Professional Efficacy	POST Professional Efficacy
Low	82 (44,8)	98 (53,6)	42 (23,0)	38 (20,8)	98 (53,6)	49 (26,8)
Moderate	50 (27,3)	32 (17,5)	57 (31,0)	66 (36,1)	32 (17,5)	31 (16,8)
High	51 (27,9)	53 (29,0)	84 (45,9)	79 (43,2)	53 (29,0)	103 (56,3)

The students most affected by burnout were those in the high EX, CY, and low PE categories. The proportion of students affected by burnout decreased in the second phase of the survey (19.67% vs. 14.75%) (Table 3).

**Table 3:** Table Distribution of responding students in the high burnout category over the two sampling periods

High level of burnout	
PRE	POST
36/183	27/183
19,67%	14,75%

### Possible predictors associated with burnout among medical and healthcare students

Students’ EX decreased except for paramedic students after the second questionnaire survey. Between the two measurements, the students’ EX score (*p* = 0.245) under quarantine decreased non-significantly. The second survey showed a significant decrease in all remaining cases compared to the first phase of the survey (*p* ≤ 0.05).

On the dimension of CY, a significant decrease was observed among men (*t* = 2.042; *p* = 0.049), those who were on medical secondment (*t* = 2.546; *p* = 0.012), including those who were hospitalized (*t* = 2.097; *p* = 0.042), or were seconded to a hospital and National Ambulance Service (*t* = 2.451; *p* = 0.001). There was also a significant decrease in the dimension of CY for students who had not had a COVID-19 infection (*t* = 3.248; *p* = 0.001) and were not under quarantine (*t* = 3.748; *p* = 0.001) and who were concerned about their health status (*t* = 2.387; *p* = 0.018).

When examining the PE dimension, there was a significant increase for physiotherapists (*t*=-3.511; *p* = 0.001) and for students who were seconded (*t*=-1.992; *p* = 0.049), who was seconded to a hospital and who were also seconded under the direction of the National Ambulance Service (*t*=-9.295; *p* = 0.001). There was also a significant improvement in the mean scores of those who were not quarantined (*t*=-2.640; *p* = 0.009) and those who were not concerned about their health status (*t*=-2.228; *p* = 0.030) (Table 4).

**Table 4:** Table Relationship between the mean scores on the maslach burnout inventory general survey for students and the examined variables

		Maslach burnout inventory – general survey for students					
		Pre-EX	Post-EX	Pre-CY	Post-CY	Pre-PE	Post-PE
Gender	*n* (%)						
Male	35 (19,1)	**9,51 (SD = 7,11)**	**7,65 (SD = 3,00)**	**7,40 (SD = 5,53)**	**4,57 (SD = 2,32)**	19,22 (SD = 5,53)	19,80 (SD = 4,10)
Female	148 (80,9)	**13,12 (SD = 7,48)**	**11,20 (SD = 6,43)**	6,77 (SD = 6,08)	6,26 (SD = 5,46)	22,79 (SD = 6,02)	23,16 (SD = 5,71)
Specialty							
Paramedic	21 (11,5)	11,14 (SD = 5,78)	12,23 (SD = 3,53)	6,28 (SD = 5,11)	7,52 (SD = 6,72)	19,95 (SD = 1,80)	21,23 (SD = 3,70)
Nurse	28 (15,3)	**14,75 (SD = 8,55)**	**11,46 (SD = 6,87)**	7,89 (SD = 6,95)	6,60 (SD = 5,56)	23,35 (SD = 7,31)	24,25 (SD = 6,60)
Physiotherapist	41 (22,4)	**11,31 (SD = 7,27)**	**10,12 (SD = 6,27)**	6,36 (SD = 5,81)	6,19 (SD = 4,51)	**22,00 (SD = 7,37)**	**23,56 (SD = 5,89)**
Laboratory analytic	14 (7,7)	**8,57 (SD = 4,60)**	**7,85 (SD = 4,84)**	4,00 (SD = 3,44)	3,57 (SD = 2,92)	25,14 (SD = 6,47)	25,57 (SD = 5,10)
Radiographer	35 (19,1)	**14,71 (SD = 8,35)**	**12,17 (SD = 7,30)**	8,37 (SD = 5,85)	6,51 (SD = 5,44)	20,74 (SD = 4,83)	20,91 (SD = 4,81)
General medicine	44 (24,0)	**12,02 (SD = 7,27)**	**9,02 (SD = 5,14)**	**6,79 (SD = 5,51)**	**4,81 (SD = 4,22)**	22,56 (SD = 5,62)	21,36 (SD = 5,47)
Assigned to medical secondment							
Yes	127 (69,4)	**13,79 (SD = 7,94)**	**11,55 (SD = 6,11)**	**8,02 (SD = 6,22)**	**6,94 (SD = 5,33)**	**20,77 (SD = 5,55)**	**21,64 (SD = 4,97)**
No	56 (30,6)	**9,33 (SD = 5,38)**	**8,19 (SD = 5,39)**	4,33 (SD = 4,14)	3,66 (SD = 3,21)	25,12 (SD = 6,19)	24,50 (SD = 6,42)
Place of medical secondment							
National Ambulance Service	76 (41,5)	**11,47 (SD = 6,59)**	**10,34 (SD = 5,58)**	6,96 (SD = 5,94)	6,48 (SD = 4,91)	22,25 (SD = 5,26)	22,14 (SD = 4,81)
Hospital	44 (24,0)	**17,88 (SD = 8,96)**	**14,40 (SD = 6,26)**	**9,81 (SD = 6,23)**	**8,15 (SD = 5,55)**	**18,06 (SD = 5,47)**	**19,65 (SD = 4,16)**
Both	7 (3,8)	**13,28 (SD = 3,90)**	**6,71 (SD = 2,92)**	**8,28 (SD = 7,31)**	**4,28 (SD = 3,71)**	**21,85 (SD = 1,95)**	**28,71 (SD = 3,90)**
Have you ever been infected with COVID-19?							
Yes	16 (8,7)	**10,37 (SD = 6,39)**	**8,62 (SD = 6,14)**	3,68 (SD = 2,62)	4,68 (SD = 3,11)	25,31 (SD = 5,16)	23,68 (SD = 4,67)
No	167 (91,3)	**12,62 (SD = 7,61)**	**10,70 (SD = 6,07)**	**7,20 (SD = 5,84)**	**6,05 (SD = 4,51)**	21,80 (SD = 6,08)	22,40 (SD = 5,67)
Have you ever been quarantined due to COVID-19?							
Yes	46 (25,1)	10,26 (SD = 7,24)	9,43 (SD = 6,75)	4,69 (SD = 4,56)	5,06 (SD = 4,91)	22,28 (SD = 5,72)	21,26 (SD = 4,90)
No	137 (74,9)	**13,16 (SD = 7,50)**	**10,89 (SD = 5,82)**	**7,63 (SD = 5,94)**	**6,23 (SD = 4,43)**	**22,05 (SD = 6,21)**	**22,94 (SD = 5,76)**
Are you concerned about your own health status?							
I’m not concerned	56 (30,6)	**9,64 (SD = 5,77)**	**8,39 (SD = 4,25)**	6,76 (SD = 4,68)	5,83 (SD = 4,90)	**19,50 (SD = 5,25)**	**21,21 (SD = 5,17)**
I’m concerned	127 (69,4)	**13,66 (SD = 7,87)**	**11,46 (SD = 6,53)**	**6,95 (SD = 6,45)**	**5,98 (SD = 5,13)**	23,25 (SD = 6,08)	23,09 (SD = 5,69)

## Discussion

Student concerns about the pandemic, and the risk of viral infection, suggest that medical and health students may be exposed to mental stress as future members of the patient care team, even when not yet present in the clinical setting permanently. At the time of our study, there were widespread concerns about the potential shortage of healthcare workers. According to the new regulations in Hungary, students studying medicine and health sciences can be assigned indefinitely without their consent. This timing allowed us to capture a truly unique perspective among students in higher education in medical and health sciences to assess the impact of secondments on student burnout scores [14].

To the best of our knowledge, the Hungarian indefinite medical secondment without consent is unique compared to the surrounding countries. Consequently, in the international literature, volunteering among medical and health science students has been the main focus of research on the current epidemic. Factors supporting willingness to volunteer included: moral responsibility (social commitment, sense of duty and caring), potential learning opportunities, personal interest, provision of appropriate personal protective equipment, parental support, expertise, knowledge, and financial compensation [18, 19].

Lazarus et al. found that the most significant demographic factors influencing willingness to volunteer were male gender, residence in the central part of the country, education in a public school, and previous volunteer activity [20].

Students who negated volunteering cited fear of infection as the main reason for not volunteering and not wanting to infect older family members living in the same household, even by accident [18].

In the past, Ádám et al. [21] and Kovács et al. [22] drew attention to the burnout of medical students through cross-sectional examinations. In their surveys, 19.7–24.5%; 1.8–27.6%; and 4.7–55.8% of students were endangered by EX; CY, and PE dimensions, respectively. Our results showed that even the dramatic changes across the country and education did not increase the degree of burnout; it reduced them. (1. Table)

In our survey, the EX scores of female respondents were found to be higher than that of male respondents, which result agrees with the survey by Győrffy et al., [23] where woman respondents were affected to a significantly greater extent in the EX dimension (32.1% vs. 41.8%, *p* = 0.012). During follow-up, there was a significant decrease in EX scores for both sexes. Male respondents were found to be more cynical in the first phase of the survey, in which value decreased significantly. Our results are consistent with those of Rusandi et al. [24] and Worly et al. [25], where EX and PE were higher in women and lower in CY than in men.

According to international literature, women face particular challenges during the COVID-19 pandemic, with fear of infection contributing significantly to stress and burnout [26, 27].

Zis et al. [28] investigated burnout among medical students before and during the COVID epidemic. Based on their results, the proportion of burned-out students did not change in the two measured periods (pre-COVID-19 18.1% vs. COVID-19 18.2%). In our study, we did not separately analyze the results of medical and health sciences students by year because the years of training and structure of the two faculties differ. Overall, our results show that the proportion of students belonging to the high burnout category decreased in the two sampling periods (19.67% vs. 14.75%).

In the sample examined by Zis et al. [28], EX decreased significantly among fourth-year students but increased significantly among sixth-year students. In contrast, the value of the CY dimension increased continuously during the training.

Studies in non-health-oriented universities have already reported the harmful effects of the coronavirus on student stress and anxiety [29, 30]. Medical students, in addition to increased mental health deterioration during the pandemic, expressed concern that the pandemic would disrupt their studies and not allow them to prepare for clinical practice [31] adequately. In Hungary, students studying medicine and health sciences were involved in medical secondments [14]. When looking at the EX dimension, the values of students studying nursing, physiotherapy, laboratory analysis, radiography, and general medicine decreased significantly. Based on the results of Ruiz et al. [32], the proportion of medical students affected by the EX dimension increased from 5 to 14.4% during the COVID period. The PE scores of physiotherapy students showed a significant decrease. The CY scores of general medicine students also showed a significant decrease. The EX and CY of paramedic students increased non-significantly but not significantly. Interestingly, students studying to become laboratory analysts were the least affected by burnout.

69.4% (*n* = 127) of the student population we studied were on a medical secondment. Students who were on medical secondment had elevated mean scores for burnout. However, it should be noted that in all cases, the mean scores for these dimensions decreased significantly in this group of students. The students most affected by student burnout were those who only completed their medical secondment in a hospital setting. It is also worth noting for this group that students’ burnout indicators improved significantly after the second data collection. It may be due to the time spent away from the university environment and the practical assignments through which students could taste the values inherent in their chosen profession.

Several publications report [33, 34] higher rates of COVID-19 infection in students in clinical settings, which sometimes negatively affect students’ mental health. At the time of our survey, coronavirus infection affected 8.7% of students. Students who had a coronavirus infection were less affected by burnout. Students who were not under quarantine (74.5%) were more affected by burnout than their peers, but the rates experienced significantly improved at the second time point of the survey.

Students concerned about their health (69.4%) had elevated mean scores on the EX and CY dimensions. For students in this group, there was a significant decrease in EX, CY, and depression at the time of the second survey.

The improved results may be because more information on COVID-19 infection and precautions has become available over time. For the second data collection period, a quasi “more prepared” audience, the education and health care sector, was waiting for students than at the beginning. By this time, the scientific community had already begun to investigate the impact of COVID-19 on mental health among healthcare staff. The negative impact of COVID-19 on stress is unquestionable, but many reports indicate that front-line combat personnel was less affected in terms of mental health. It should be noted, however, that the international literature could be more consistent, as the severity and rapidity of infection waves varied between/across national borders.

Numerous publications substantiate [35–37] that resilience and hypermentalizing reduce the depression, anxiety, and stress levels of healthcare workers, thus resulting in lower burnout rates. Educational and training programs should consider these connections, especially during pandemic situations. Additionally, during COVID-19, depression, anxiety, and stress levels were significant in the population, mostly associated with factors such as smoking habits, fear of illness, religious beliefs, and socio-economic status. To protect the mental health of healthcare workers and mitigate the impact of the pandemic, it is necessary to monitor the effects and put in place coping techniques and emergency measures.

### Limitations

One of the limitations of our study was the low response rate, which was assumed to be due to the challenges faced by the students. Some students may have stopped reading the newsletter due to the workload. Another significant proportion of students presumably struggled with personal challenges related to the pandemic. It should also be noted that our survey focused only on students studying medicine and health sciences at the University of Pécs. We tried to increase the response rate by making our questionnaire available on social media platforms and involving representatives of the relevant specializations, including sending reminders for completion.

## Conclusions

Our study, although based on a relatively small number of student responses, focused on the pandemic period, including healthcare deployments and the experience during the coronavirus infections, provides preliminary results and considerations for future research. Further insights are needed on the short- and long-term impact of the COVID-19 pandemic on medical and health science students and their education. Future studies need to target long-term and region-specific assessment of burnout rates and how students tolerate long-term medical and health science education disruptions.

In conclusion, medical secondments positively affected student burnout scores for students studying medicine and health sciences at our institution. This fact implies that it is necessary to have more internships in real-life settings during the training, which would increase the student’s sense of personal efficacy. Given the relatively low number of items, our results are indicative, and no direct conclusions can be drawn. Further targeted studies are recommended to understand the reasons behind the observed values further to understand the relationships better.

## Data Availability

The datasets used and analyzed during the current study are available from the corresponding author upon reasonable request. (DS)

## List of abbreviations

MBI: GS (S)–Maslach Burnout Inventory General Survey for Students
EX: Exhaustion
CY: Cynicism
PE: Professional Efficacy
